# Epidemiology and factors associated with the perioperative course of patients undergoing hip fracture during the initial phase of the state of emergency declared in 2020

**DOI:** 10.3389/fmed.2025.1473619

**Published:** 2025-05-12

**Authors:** S. Santander, A. Lanuza, J. Longás, F. Úbeda, C. Marco, M. J. Luesma

**Affiliations:** ^1^Department of Pharmacology, Physiology, and Legal Forensic Medicine, Faculty of Health and Sports Sciences, University of Zaragoza, Huesca, Spain; ^2^HCU Lozano Blesa, Zaragoza, Spain; ^3^HCU Miguel Servet, Zaragoza, Spain; ^4^Mérida Hospital Complex, Mérida, Spain; ^5^Department of Human Anatomy and Histology, Faculty of Medicine, University of Zaragoza, Huesca, Spain

**Keywords:** hip fracture, gamma nail, anesthesia, locoregional technique, COVID-19, morbidity and mortality

## Abstract

**Introduction:**

Currently, there is no gold standard anesthetic plan for elderly patients with hip fractures who must undergo surgery. The state of alarm during 2020 due to the COVID-19 pandemic changed certain hospital paradigms, prompting an investigation into differences in anesthetic management and patient survival for hip fracture surgeries using gamma nail osteosynthesis in 2020 compared to 2019.

**Materials and methods:**

A historical cohort study was conducted to assess morbidity and mortality, with data obtained from anonymized medical records.

**Results:**

Statistically significant differences were found, notably an increase in Body Mass Index during confinement. A statistically significant decrease in leukocyte and hemoglobin levels was observed after surgery. However, there were no statistically significant differences in mortality between 2019 and 2020. Patients who did not require vasoactive drugs during surgery to maintain blood pressure levels above 90/60 mmHg had a better survival rate.

**Conclusion:**

Despite the reduction in postoperative hospital surveillance time, no increase in complications was found. This allowed for early patient reintegration into daily life, early functional rehabilitation, and the minimization of economic costs.

## 1 Introduction

Hip fracture is a condition whose incidence increases exponentially from the sixth decade of life. In the elderly population, the pathogenesis of hip fractures is typically multifactorial, with several identifiable risk factors: low bone quality with decreased bone mineral density, high likelihood of falls, dementia, poor functional status, neuromuscular changes, polypharmacy, and multiple comorbidities (1–3). In Spain, the incidence is estimated at 104 cases per 100,000 inhabitants, amounting to between 45,000 and 50,000 hip fractures per year (4).

Most hip fractures occur at home during activities of daily living (1–4). Therefore, it was anticipated that their incidence would not decrease despite the social isolation policies implemented during the onset of the State of Alarm in Spain in 2020 due to the SARS-CoV-2 infection.

Elderly patients often present with a constellation of pre-existing conditions that significantly influence prognosis and recovery following surgery. The Charlson Comorbidity Index (CCI, see Annex A) assigns weighted scores to these comorbidities, providing an estimate of life expectancy based on the cumulative score (5). The CCI serves as an easily applicable, rapid, and cost-effective evaluation method, demonstrated to be a reliable predictor of 10-year mortality (1, 6, 7).

The ASA score (American Society of Anesthesiologists, see Annex B) stands as the prevailing standard for assessing perioperative anesthetic risk and estimating life expectancy based on patient comorbidities. Presently, it is universally employed during preanesthetic evaluations (5, 8).

Despite advancements, there remains a dearth of scientific evidence to advocate for a definitive gold standard anesthetic approach for elderly patients with hip fractures (9). Both general anesthesia (GA) and locoregional anesthesia (LRA) represent safe and efficacious options for this demographic undergoing hip fracture surgery (10). Studies indicate that neuraxial anesthesia offers certain advantages, particularly highlighted amid the current pandemic: it obviates airway management and aerosol generation, negating the need for orotracheal intubation and even enhancing postoperative respiratory function and dynamics. Conversely, general anesthesia ensures a more stable hemodynamic profile and circumvents complications associated with neuraxial techniques, such as epidural hematoma (11, 12).

The Anesthesiology Societies emphasize the crucial role of expert anesthesiologists in determining the appropriate anesthesia type, taking into account the patient’s pre-anesthetic evaluation, comorbidities, and potential complications, alongside their own clinical expertise (12–14).

Postoperative complications, including anemia, infection, delirium, and psychomotor agitation, are prevalent in the natural course of hip fractures, particularly among patients aged 65 and above, with incidence rates ranging between 20 and 50% according to various studies. Increased mortality rates in these post-surgery patients have been linked to factors such as advanced age, presence of comorbidities, pre-existing functional status, and individualized surgical plans (15–18).

In light of the SARS-CoV-2 pandemic, clinical practice has undergone significant adaptation to navigate complex circumstances. These adaptations have led to prolonged delays in surgical intervention, increased reliance on blood transfusions and vasoactive medications, shortened hospital stays, shifts in anesthetic techniques chosen by healthcare professionals, and ultimately, heightened morbidity and mortality rates among affected patients compared to those managed similarly in the preceding year.

Several hospitals have conducted studies on the impact of COVID-19 on their clinical practice. There has been a reduction in surgical activity since the beginning of the pandemic, affecting both elective and emergency procedures. Some examples illustrating this situation include Hospital Dr. Josep Trueta, which experienced a 93.8% decrease in elective surgical activity and a 72.7% reduction in emergency surgeries during the first 4 months of the pandemic. Similarly, Hospital Universitario de la Princesa in Madrid suspended almost all surgical activity within the first month (19, 20). Like other surgical specialties, in trauma services the number of interventions declined. For instance, at Hospital Universitario Arnau de Vilanova in Lleida, a study evaluating surgical delays in spinal procedures revealed that the number of spine surgeries decreased by 32.15% in the first year of the pandemic compared to the previous year (21). Hospitalization length in Orthopedic Surgery and Traumatology services was also affected. At the Traumatology Department of Hospital Universitario de Canarias, Tenerife, the average postoperative hospital stay decreased. Prior to the lockdown, patients were hospitalized for an average of 11 days, whereas post-lockdown, the average stay was reduced to 4 days. In the same department, the average postoperative stay for hip surgery, which was previously 15.35 days, was reduced to approximately 1 week after the lockdown (22).

These developments prompt a new avenue of inquiry, focusing on how the management of geriatric surgical patients during this pandemic has impacted perioperative complications, morbidity, and mortality rates (23). Specifically, our investigation aims to analyze data collected from the Lozano Blesa University Clinical Hospital (HCU) in Zaragoza regarding the management of hip fractures during the State of Alarm, comparing it with data from the corresponding months of 2019. Through this analysis, we seek to identify statistically significant differences in the time elapsed from fracture to surgical intervention, duration of postoperative hospitalization, and incidence of post-surgical complications graded according to the Clavien and Dindo scale (see Annex C).

## 2 Materials and methods

An analytical, observational, and retrospective study of historical cohorts of patients undergoing hip fracture surgery was conducted at HCU Lozano Blesa. Data were obtained through the review of medical records, ensuring the anonymization of personal information. The study was performed in accordance with the ethical standards of the Declaration of Helsinki and received approval from the Research Ethics Committee of the Autonomous Community of Aragon (CEICA) (Minute No. 10/2021; CP - CI PI21/257). As this was a retrospective study with anonymized data and access to medical records authorized by the Medical Directorate, the preparation of a patient information document or informed consent was deemed unnecessary, in accordance with the exceptions allowed by CEICA for such documents.

The medical records of patients who underwent hip fracture surgery using a gamma nail were reviewed during the initial period of the State of Alarm (03/15/2020 to 06/15/2020), which is representative of the pandemic situation. Only patients with negative PCR results for SARS-CoV-2 or those free of COVID-19 symptoms were considered, as systematic testing was not yet universally available. These patients were compared with a control group of patients who underwent the same surgical procedure during the same dates in 2019, a year with regular surgical activity. By retrospectively comparing these groups, we investigated the relationship between various factors (demographic, clinical, anesthetic, and surgical) and intraoperative complications, morbidity and mortality, and one-year survival rates.

The gamma nail was chosen as a treatment option during the pandemic due to its surgical efficiency. It is important to clarify the selection of the gamma nail as the preferred method for the fixation of extracapsular femoral fractures, replacing traditional options such as the DHS plate due to its multiple biomechanical and clinical advantages (24). Its design allows for better force distribution along the femur, thereby decreasing postoperative complications and the need for additional interventions. Furthermore, it is particularly beneficial for elderly patients, who tend to be more vulnerable to respiratory complication (25). Compared to the DHS plate, the gamma nail reduces the risk of secondary fracture displacement, decreases the likelihood of nonunion, minimizes intraoperative blood loss, and shortens surgical time and perioperative morbidity so minimizes the risk of COVID-19 exposure. However, it is not exempt from risks, including the potential for distal femoral fracture due to its levering effect or the risk of cephalic screw penetration into the femoral head (24, 25).

Inclusion criteria:

- Deferred emergency surgery or scheduled surgery
- Extracapsular femur fractures
- Osteosynthesis surgery using short or long gamma nails
- Locoregional anesthetic technique: intradural anesthesia with or without peripheral nerve block using ultrasound-guided technique
- Local anesthetic used in the subarachnoid space: hyperbaric bupivacaine in various doses

Exclusion criteria:

- Intracapsular fractures, fractures of the femoral shaft, or distal third of the femur
- Conservative fracture management
- Time to seek healthcare greater than 3 days after the trauma causing the hip fracture
- Osteosynthesis surgery using plate and screws, total or partial hip prosthesis
- General anesthesia
- Local anesthetic used in the subarachnoid space: levo-bupivacaine, isobaric bupivacaine
- ASA IV patients due to their short life expectancy after surgery and complex baseline conditions, as these would affect sample homogeneity and introduce extreme values
- Age < 65 years
- Multiple trauma patients
- Positive SARS-CoV-2 PCR (year 2020), as a considerable increase in mortality has been demonstrated compared to patients with negative SARS-CoV-2 PCR

The statistical analysis was performed using IBM® SPSS Statistics software, Version 25.

A total of 57 patients who underwent hip fracture surgery were studied between March 15 and June 15 in the years 2019 and 2020. Normality tests were conducted to assess the distribution of the quantitative variables included in the study. The Kolmogorov–Smirnov test was used as the sample size was *N* = 57 patients (*N* > 50).

For the management of missing values, these were defined by the researchers as data not obtained for the dependent variables. Consequently, the analysis was based on cases without missing values for any dependent variable or factor included in the study.

Descriptive statistics were first conducted to understand and verify the characteristics of the selected sample (*N* = 57). In hypothesis testing, differences with *p*-values < 0.05 were considered statistically significant.

Survival was defined as the time until the event of interest occurred, in this case, the death of a patient due to etiologies related to the care, surgical, and anesthetic processes of the pathology. Patients who did not experience the event (death) or who died from causes unrelated to the traumatological process under study were considered censored; examples include deaths from neoplasms.

To conduct the survival analysis, which was treated as a qualitative variable (year 2019 versus year 2020) and the time elapsed until another qualitative variable (survival), descriptive statistics were analyzed using Kaplan–Meier curves. To determine if the differences were statistically significant, the curves were compared using the Log-Rank test.

## 3 Results

### 3.1 Descriptive statistics

A total of 57 patients who underwent hip fracture surgery between March 15 and June 15 in the years 2019 and 2020 were studied. Among the selected patients, 24.6% were men (14/57) and 75.4% were women (43/57), with an average age of 87 years ± 8. The average BMI (Body Mass Index) was 26.6 ± 5.5.

Regarding the abbreviated Charlson Comorbidity Index, 40.4% of patients (23/57) had no comorbidities (scores of 0–1), 22.8% (13/57) had low comorbidity (score of 2), and 36.9% (21/57) had high comorbidity (scores of 3–4). The ASA classification to assess anesthetic risk was as follows: 0% ASA I (no patients in this category), 54.4% ASA II (31/57), 45.6% ASA III (26/57), and 0% ASA IV (excluded based on the previously mentioned criteria).

In 49% of the patients (28/57), one or more transfusions of packed red blood cells were administered perioperatively to achieve the recommended hemoglobin values.

63% of patients (36/57) experienced at least one episode of intraoperative hypotension (defined as systolic arterial pressure < 90 mmHg and/or diastolic blood pressure < 60 mmHg), with 97% of these cases requiring vasoactive drugs (ephedrine or phenylephrine) to restore blood pressure levels.

Locoregional anesthesia techniques were administered to all patients in the study. The dose of 0.5% hyperbaric bupivacaine had a median of 9 mg ± 2. Additionally, in 67% of the patients (38/57), this technique was complemented by a peripheral nerve block (femoral and femoral cutaneous nerves), performed in an ultrasound-guided manner with the administration of 0.25% levo-bupivacaine (15–30 cc).

The study also evaluated the surgical delay time, defined as the hours that elapsed from the patient’s initial contact with the healthcare system following the fall to the surgical intervention. The average surgical delay for the overall sample was 44 h ± 4.

Regarding postoperative complications, the most frequent were psychomotor agitation and anemia, each occurring in 45.6% of the patients (26/57).

The next most common complication was suboptimal pain control, which occurred in 31.6% of patients, as they frequently requested additional analgesics. The relationship between postoperative pain control and the presence of a peripheral nerve block during the anesthetic procedure was evaluated. In the group without a peripheral nerve block, 63% of patients (12/19) experienced poor pain control postoperatively. This percentage was significantly reduced in the group that received an ultrasound-guided peripheral nerve block with the administration of local anesthetic at the level of the femoral and femoral cutaneous nerves, where only 16% of the patients (6/38) requested additional analgesia. These differences were statistically significant (chi-square = 13.154; *p* < 0.01).

When examining mortality during hospital admission, 6 out of 57 patients died (10%). One year after the surgery, 13 out of 57 patients had died (23%). Regarding overall mortality, the primary cause of death among the study patients was nosocomial pneumonia (56%), followed by decompensation of pre-existing heart failure (31%) and sepsis of urinary origin (6%). It is noteworthy that at the Lozano Blesa HCU, mortality within the first year after surgery ranged from 15% to 27%.

### 3.2 Hypothesis contrast

A comparative analysis was conducted between the proposed groups (surgical interventions performed in 2019 vs. 2020). In 2019, 76% (28/37) of patients undergoing hip fracture surgery were women. A similar proportion was observed in 2020, with 75% (15/20) being women. No statistically significant differences were found regarding sex distribution.

**Figure 1:**
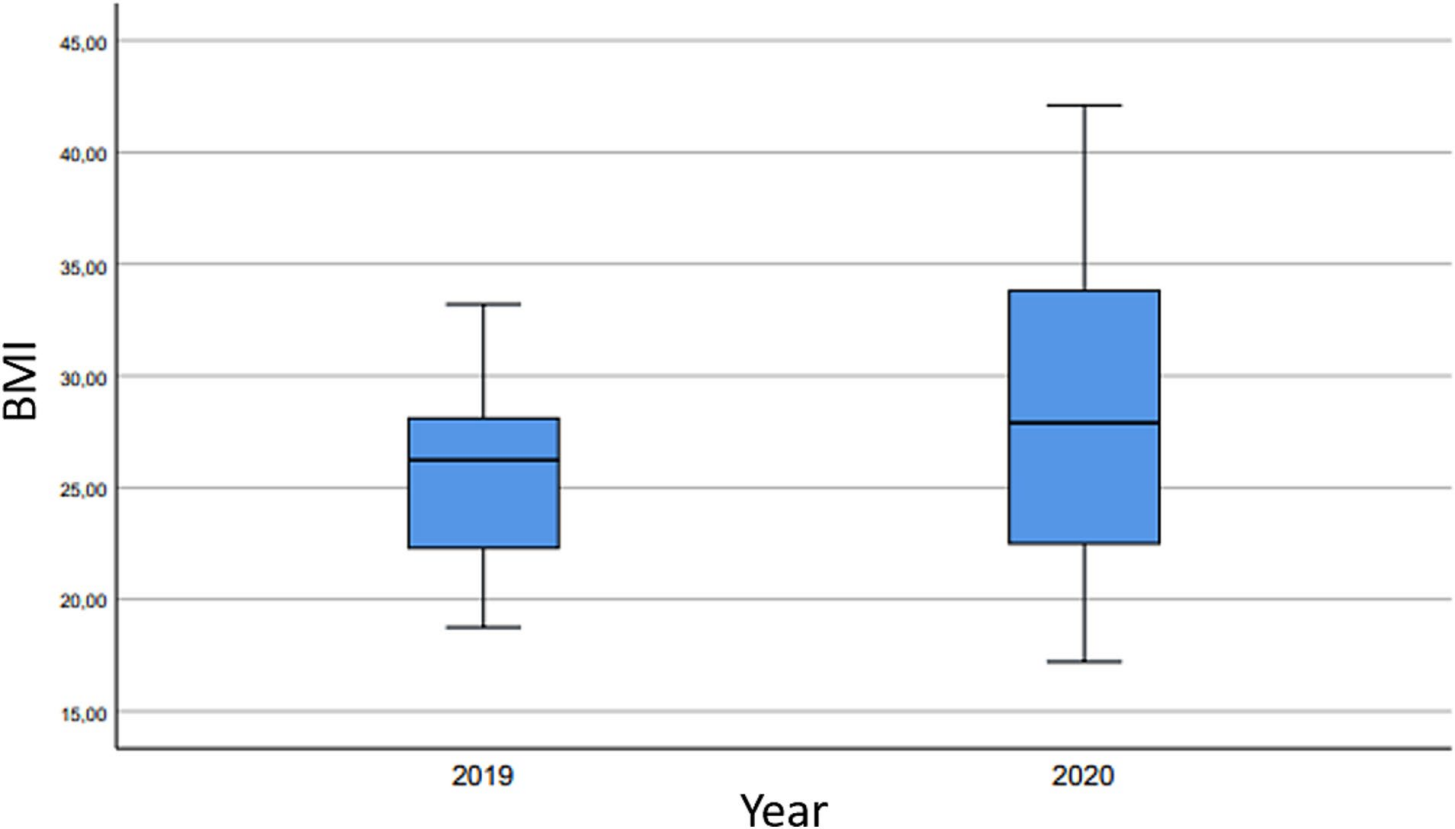
Body mass index (BMI)—increase in average BMI value and dispersion measures. In 2019, the minimum BMI recorded was 18.75, while the maximum was 33.20. Comparatively, in 2020, the minimum BMI was 17.21, and the maximum was 42.10.

Regarding BMI, the average value in 2019 was 25.64 ± 3.75, while in 2020 it was 28.80 ± 7.67 (Figure 1). These differences were statistically significant, as indicated by the student’s t-test [t = −2.24; 95% CI: (−6.47, −0.33); *p* = 0.03], showing an increase in BMI during the confinement and State of Alarm decreed in 2020.

For analytical variables, the perioperative variation in leukocyte and hemoglobin levels was examined by comparing the baseline figures before surgery with the analytical control obtained 48 h post-surgery. Upon applying the student’s t-test for paired data, differences were observed in both leukocyte count (t = 2.574; 95% CI: 2252.45–280.88; *p* = 0.01) and hemoglobin count (t = 9.201; 95% CI: 3.135–1.703; *p* < 0.01). Thus, a statistically significant decrease in leukocyte and hemoglobin levels was noted after surgery.

The study also evaluated the hours of surgical delay, defined as the time elapsed since the patient’s initial contact with the healthcare system after the fall and surgical intervention. The median hours of surgical delay were 48 h in 2019 and 24 h in 2020. Although this variable did not follow a normal distribution, the Mann–Whitney U test was applied, revealing no statistically significant differences between the two time periods (U = 257,500; *p* = 0.05).

In terms of hospital admission duration, the median admission was 7.0 ± 36.0 days in 2019 and 4.5 ± 2.0 days in 2020. These differences were statistically significant (U = 166; *p* < 0.01), indicating shorter admission times in 2020.

Locoregional anesthesia techniques were administered to all patients in the study, with the dose of 0.5% hyperbaric bupivacaine yielding a median of 9 ± 3 mg in 2019, compared to 10 ± 1 mg in 2020. Notably, peripheral nerve blocks were performed on 76% (28/37) of patients in 2019, while this figure decreased to 50% (10/20) in 2020. However, these differences were not statistically significant.

Regarding postoperative pain control, in the group without peripheral nerve block, 63% of patients (12) experienced inadequate pain management during the postoperative period. In contrast, among those who received ultrasound-guided peripheral nerve block with local anesthetic administration at the femoral and femoral cutaneous nerve levels, only 16% of patients (6) required additional analgesia. These differences were statistically significant (chi-square = 13.154; bilateral asymptotic sig. < 0.01).

Statistically significant differences were observed (chi-square = 4.023; *p* < 0.05) in favor of patients who did not require intraoperative vasoactive drugs, with a mean survival of 344 days (95% CI: 312.8–375.3), compared to those who needed them for maintaining blood pressure control, with a mean survival of 269 days (95% CI: 218.4–319.9).

Regarding mortality during admission, 5 out of 37 patients died in 2019 (13%), while only 1 out of 20 patients died in 2020 (5%). One year after surgery, 10 out of 37 patients operated on in 2019 died (27%), whereas this number decreased to 3 out of 20 patients in 2020 (15%). However, no statistically significant differences were found when applying the Fisher exact test to compare mortality rates at admission or 1 year after surgery between 2019 and 2020.

No statistically significant differences were observed for the remaining variables included in the study.

### 3.3 Survival analysis

**Figure 2:**
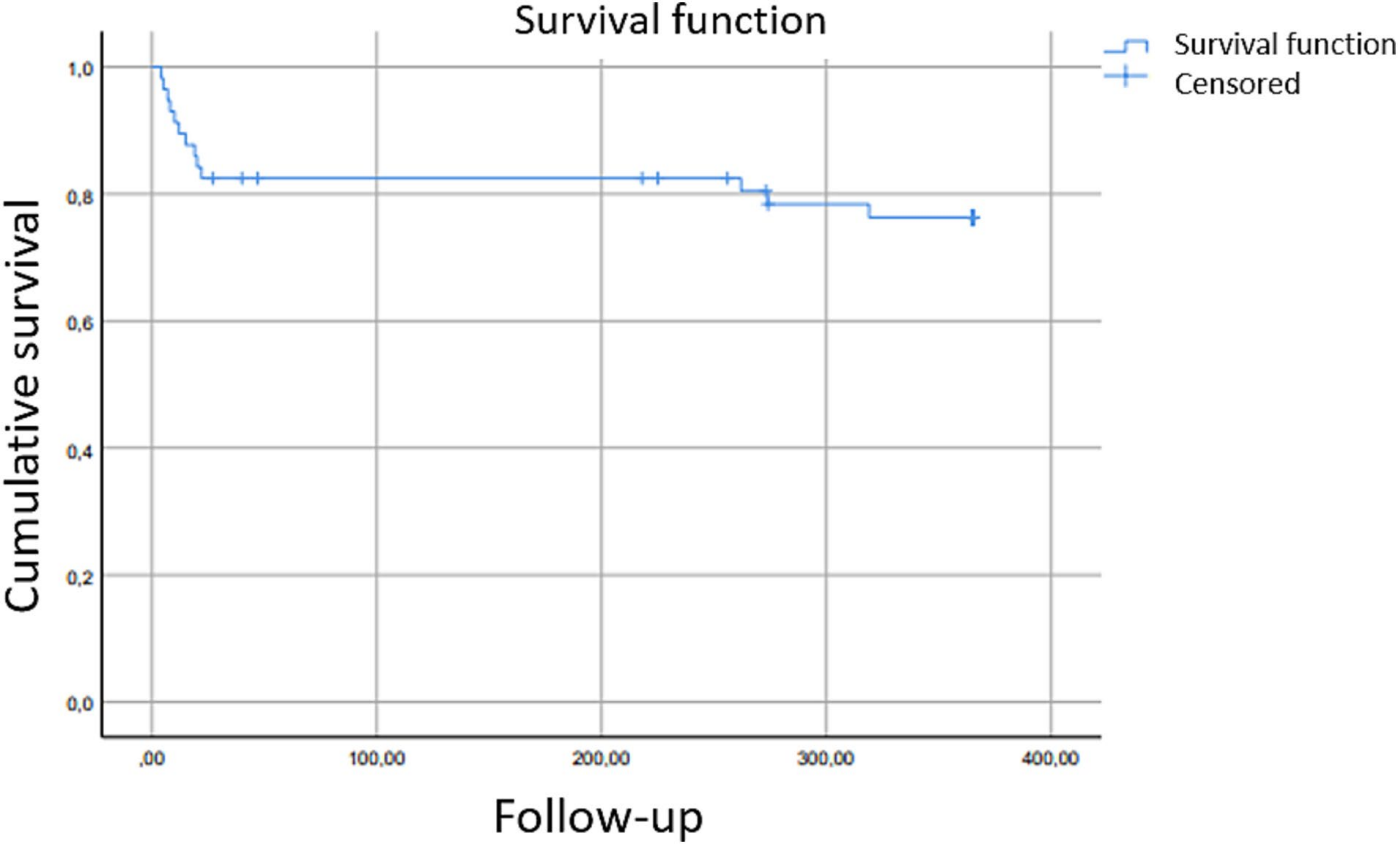
Kaplan–Meier curve. The curve illustrates the two periods with the highest mortality following hip fracture surgery. Notably, the timing of hospital admission becomes increasingly relevant as the year post-surgery progresses. Patients were followed for 365 days.

When conducting the overall survival analysis using the Kaplan–Meier method (Figure 2), we observed an overall mortality rate of 22.81%. However, it was not possible to calculate the median survival as it was not reached within the timeframe of this study.

When separating and study mortality in the selected groups (year 2019 vs. year 2020), the Long-Rank test was used to compare the Kaplan–Meier curves obtained (Table 1).

**Table 1:** Long-rank test results to compare the obtained Kaplan–Meier curves.

			Censored	
Year	Total *N*	*N* of events	*N*	Percentage
2019	37	10	27	73.0%
2020	20	3	17	85.0%
Global	57	13	44	77.2%

			*Mean*	
			*95% confidence interval*	
Year	Estimation	Standard error	Lower limit	Upper limit
2019	283.314	23.639	236.984	329.644
2020	325.850	23.922	278.963	372.737
Global	298.182	17.708	263.475	332.890

When examining 1-year survival rates following surgery, 10 patients operated on in 2019 (27%) and 3 patients in 2020 (15%) passed away. The mean survival duration in 2019 was 283.3 days [95% CI: 236.9–329.6], increasing to 325.8 days [95% CI: 278.9–372.7] in 2020. Due to more than 50% of patients surviving beyond this period, the median survival could not be calculated.

**Figure 3:**
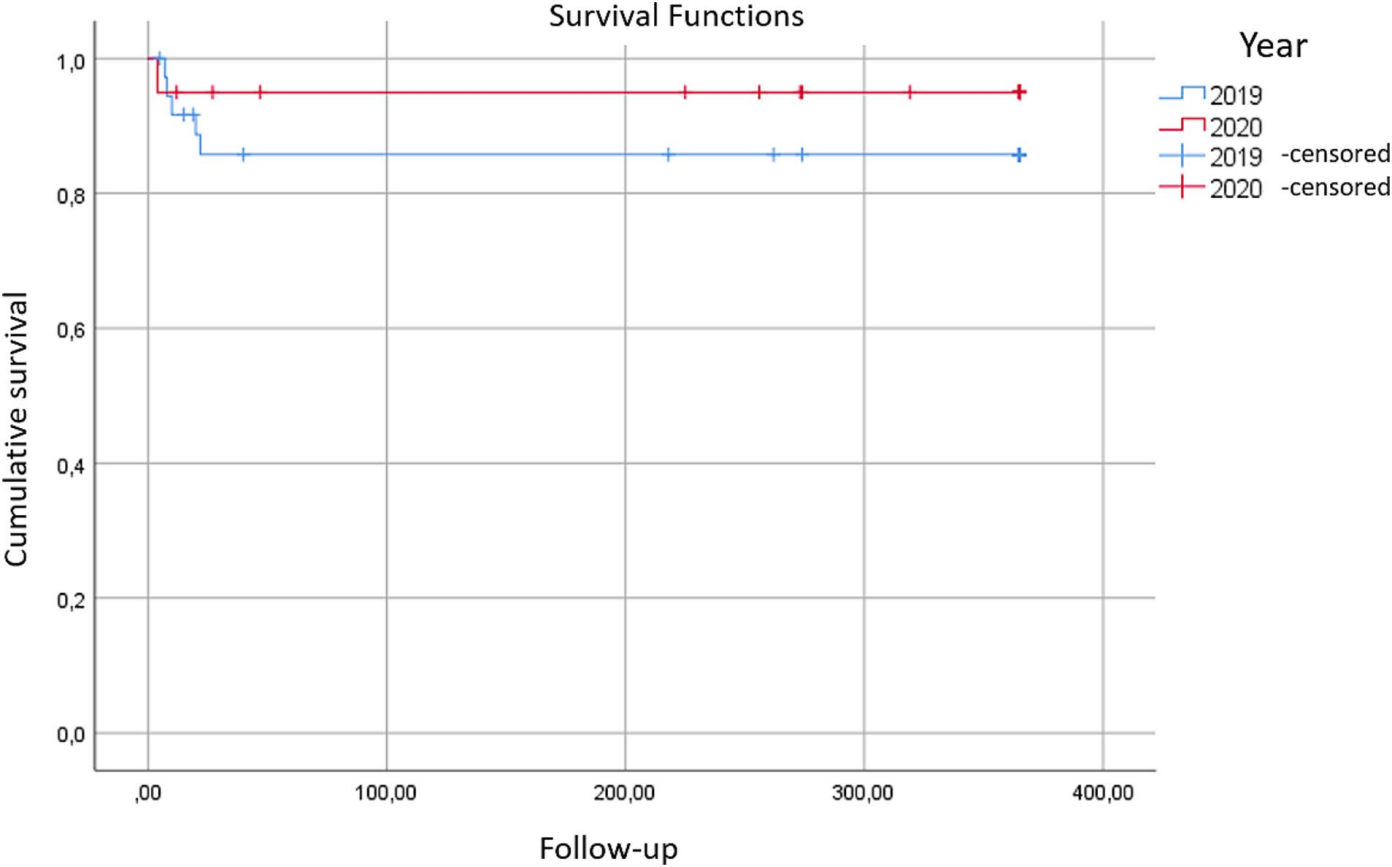
Kaplan–Meier curves for the year 2019 vs. the year 2020 illustrate the survival rates of hip fracture patients at the Hospital Clínico Universitario Lozano Blesa during the months of March to June. No statistically significant differences were detected in mortality between these periods, despite the presence of the state of alarm due to COVID-19 in 2020.

When comparing the survival curves using the Long-Rank test, we obtained a chi-square value of 0.828 (*p* = 0.36). Therefore, the differences in mortality rates between patients who underwent surgery in 2019 and those who underwent surgery in 2020 were not statistically significant (Figure 3).

**Figure 4:**
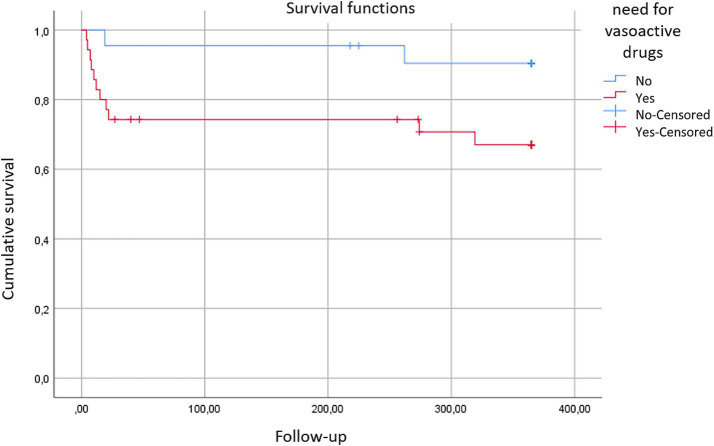
Survival curves: these curves illustrate the survival outcomes based on the necessity for vasoactive drugs (red) vs. those not requiring them (blue) to maintain adequate blood pressure control during the intraoperative period.

When examining the anesthetic variables included in the study, statistically significant differences in survival were observed (Long-Rank, chi-square = 4.02, *p* < 0.05). These differences favored patients who did not require vasoactive drugs during surgery to maintain blood pressure levels above 90/60 mmHg, with a mean survival of 344 days (95% CI: 312.9–375.3), compared to those who did require them, with a mean survival of 269 days (95% CI: 218.4–319.9) (Figure 4).

To explore the impact of various variables on patient survival, data analysis was conducted using Cox Regression. The results revealed that for each additional point on the Clavien Dindo scale, the annual mortality rate increased by 2.5 times in a statistically significant manner (*p* < 0.01). Likewise, for every 1 g/dL decrease in hemoglobin levels in the post-surgical blood analysis at 48 h compared to the preoperative levels, there was a corresponding 1.6 times higher mortality rate (*p* = 0.03) (Table 2).

**Table 2:** Multivariate Cox Regression analysis.

	B	SE	Wald	df	Exp(B)
Step 1: Clavien Dindo	0.927	0.229	16.351	0.000	2.526
Step 2: Clavien Dindo	1.252	0.306	16.706	0.000	3.498
Hb post-sg, control	0.466	0.220	4.503	0.034	1.594

Significant differences were observed regarding the impact on 1-year survival based on the score obtained on the Clavien Dindo scale during admission and postoperative hemoglobin levels. For each additional point in these variables, the likelihood of survival decreased by 2.5 times and 1.6 times, respectively. Hb post-sg, Hemoglobin post-surgery. B: regression coefficient; SE: Standard Error of the coefficient B; Wald: Wald statistic; df: This represents the degrees of freedom; Exp(B): exponentiation of the coefficient B.

## 4 Discussion

Veronese et al. (1) and Lamb et al. (2) advocate for maintaining the monitoring of hip fracture incidence during the State of Alarm, including the period of confinement mandated by the Government of Spain between March 15 and May 15, 2020. Accidental falls at home are the most common cause of hip fractures in the elderly. However, consensus is lacking, and it is logical to anticipate a decrease in this pathology due to reduced physical activity and social mobility among these patients.

The National Registry of Hip Fractures (3) suggests that reduced activity among the elderly, coupled with increased support from younger relatives at home, could result in more attentive care and a lower incidence of hip fractures. In our study, the number of patients requiring hip fracture surgery during the same time period in 2019 and 2020 decreased from 37 to 20 patients. Expanding on these findings, a total of 116 hip fracture surgical interventions were recorded throughout the entire year of 2019, and 104 in 2020. While no statistically significant differences were identified, these data hint at a potential reduction in hip fracture incidence during the State of Emergency at the HCU Lozano Blesa.

Regarding days of hospital admission, the median duration was 7.0 days in 2019 and 4.5 days in 2020, representing a statistically significant difference. This reduction helps avoid unnecessary prolongation of hospital stays and decreases the risk of post-surgical complications and nosocomial infections, specifically SARS-CoV-2 infection in the hospital environment. Despite the shorter hospital surveillance time postoperatively, no increase in complications was observed. This facilitates early patient reintegration into daily life and prompt functional rehabilitation, while also minimizing economic costs. An appropriate discharge plan, along with close follow-up and proper education on post-operative care, can improve both functional outcomes and the satisfaction of the patient and their family environment (26).

In terms of 1-year survival following surgery, the number of deaths among patients operated on in 2019 was 10 (27%), whereas in 2020, this decreased to 3 patients (15%). Similarly, the mean survival duration in 2019 was 283.3 days [95% CI: 236.9–329.6], compared to 325.8 days [95% CI: 278.9–372.7] in 2020. Although no statistically significant differences were detected, this may be attributed to the small sample size.

The delay in surgical time has been recognized as an independent factor contributing to increased post-surgical mortality (27). In our study, the median surgical delay was 48 h in 2019 and 24 h in 2020, aligning with the current recommendations (<48 h) outlined in the Guide for the Management of Hip Fractures by Griffiths et al. (28). However, despite meeting these recommendations, no statistically significant differences were observed. Given the limited sample size of this study, further research is warranted to investigate whether the surgical delay time was indeed shorter in 2020, as suggested by our data. Despite the strain on healthcare resources, our findings indicate the potential for optimizing resource management and reducing hospital stays associated with this pathology.

Assessing the baseline situation and nutritional status of patients is crucial preoperatively. Malnutrition remains a significant concern in geriatric research due to its high prevalence and detrimental impact on functional recovery, mortality rates, and healthcare costs. Elderly patients with pre-existing malnutrition before hip fracture surgery often exhibit worsened functional status, increased morbidity and mortality, prolonged hospital stays, and poorer post-surgical functional outcomes (29).

Although Bohl et al. (30) reported that factors such as lack of exercise, reduced access to nutritious foods, and psychological stress during the pandemic contributed to a decreased intake of healthy foods and an increased consumption of calorie-dense processed foods high in saturated fats, leading to a more pronounced prevalence of malnutrition among elderly patients compared to previous years, this trend was not observed in the same manner in Spain. In Spain, the culinary culture is based on the Mediterranean diet, which supports adequate adherence to nutritional requirements. The Spanish Society of Community Nutrition (SENC), in collaboration with various Spanish universities, conducted a cross-sectional study analyzing the hygienic-dietary habits of the Spanish population during the pandemic. According to the study, the regular consumption of canned foods, cookies, pastries, chocolate, and sugary beverages decreased. These findings are supported by reports from distribution chains, which documented a decline in sales of pastries, cookies, and snacks. The primary issue appears to have been the reduced capacity to engage in physical exercise during the lockdown. Overall, physical activity levels decreased; among those who exercised, 15% stopped entirely, while an additional 14% reported engaging in exercise only occasionally. Another factor affecting the population’s baseline health status was sedentary behavior, more than 60% of individuals spent five or more hours sitting daily, and 25% spent nine or more hours. Additionally, among smokers, 30% reported an increase in tobacco consumption. Sleep quality was also impacted, with up to 37% of individuals reporting poor sleep during the lockdown (31). These data suggest that despite an adequate diet, sedentary behavior and reduced sleep hygiene increased the risk of malnutrition, as the balance between caloric intake and physical activity became disproportionate. This supports the observation of a significant increase in BMI within the population during the lockdown.

Indeed, in our study cohort, statistically significant differences were observed in the average BMI of patients between 2019 and 2020, with values increasing from 25.64 (indicating close to normal weight) to 28.80 (suggesting overweight status), respectively.

Frailty, an emerging concept gaining importance in clinical practice (32), lacks consensus in its definition, diagnosis, and clinical aspects. According to Lim et al., frailty assessment serves as a valuable risk stratification tool in preoperative anesthetic evaluation (33, 34). The ASA classification is widely used to assess perioperative risk, but it may not fully capture the complexity of frailty in elderly patients. Tools such as the Clinical Frailty Scale (CFS) and the Edmonton Frailty Scale (EFS) have been developed to assess frailty more specifically. However, these scales were not evaluated in our patient cohort. Integrating these scales into the preoperative assessment could enable better identification of at-risk patients, facilitating personalized interventions and more appropriate anesthetic planning (35, 36).

In our study, no statistically significant differences were observed in the levels of inflammatory parameters associated with Frailty Syndrome in 2020 compared to the previous year. Changes in inflammatory status resulting from lifestyle modifications typically manifest gradually over months or years rather than weeks. In the referenced study, no significant differences were observed in 2020 compared to 2019, possibly because the observation period (March–June 2020, the early phase of the pandemic) was too short to capture physiological changes in blood tests. External evidence supports this hypothesis; for instance, a lifestyle improvement program required 12 months to achieve significant reductions in high-sensitivity C-reactive protein among older adults, whereas shorter durations showed minimal changes (37). This suggests that inflammatory markers respond slowly to interventions or alterations in physical activity and nutrition, thus a period of only 2–3 months might not be sufficient to produce detectable changes in frail patients.

During 2020, COVID-19 restrictions induced drastic lifestyle changes potentially influencing chronic inflammation, particularly in elderly and frail populations. Many older adults experienced decreased physical activity and increased sedentary behavior due to lockdown measures (closure of day centers, gyms, and enforced distancing). Such inactivity can promote loss of muscle mass and functional capacity; even brief immobilization in older individuals results in muscle atrophy and insulin resistance, conditions associated with subclinical inflammation. Furthermore, dietary habits were significantly altered. Multinational studies indicate that during lockdowns many individuals increased their intake of unhealthy foods (snacks, ultra-processed foods) and calories, accompanied by greater meal frequency. In vulnerable populations, this often translated into weight gain (especially increased fat mass) or covert malnutrition, exacerbating pre-existing frailty (38). Although it has already been mentioned this trend was not observed in the same manner in Spain because of the Mediterranean diet (31). Additionally, pandemic-related psychosocial stress and isolation could affect inflammatory pathways. Social distancing and fear of the virus heightened anxiety and depression levels among many older adults. Chronic stress can disrupt neuroendocrine responses; for instance, geriatric residents in lockdown demonstrated abnormal cortisol patterns (marked morning decreases and flattened daily rhythms), attributed to prolonged stress and loneliness (39).

Traditionally, preventive and therapeutic efforts have focused on postoperative functional rehabilitation. However, recent research suggests that the immediate postoperative period may not be optimal for such interventions. Patients often experience fatigue and pain, hindering active engagement in their recovery. Consequently, concepts such as prehabilitation and frailty are gaining prominence, emphasizing interventions during the preoperative period when patients typically possess greater physical strength and emotional stability. Prehabilitation aims to enhance patients’ functional capacity, minimize emotional stress, and optimize their nutritional status and management of pre-existing conditions.

The rise in life expectancy and the aging of the population have led to a notable shift in the population pyramid, characterized by a progressive increase in age groups over 75 and 80 years old, as evidenced by annual census data in Spain. This trend is reflected in our study, where the average age of participants was 87 years. This demographic shift has led to an increased prevalence of surgical treatments for conditions common in these older age brackets, often involving fragile patients.

However, age alone is not the sole factor contributing to perioperative risk. Research indicates that other factors, such as comorbidities and the patient’s preoperative functional status, play significant roles in post-surgical outcomes.

Preoperative prehabilitation—multimodal interventions (exercise, nutrition, psychological support) implemented prior to surgery to enhance patient resilience—became especially relevant during the pandemic but faced unprecedented challenges. Due to cancelations or delays in elective surgeries and the need to protect vulnerable patients, healthcare teams had to adapt prehabilitation strategies to remote formats (40). Traditional face-to-face sessions were replaced by tele-prehabilitation programs to keep patients active and prepared while awaiting surgery. Practical adaptations included: Remotely supervised exercise (home-based exercise routines were implemented. For very frail patients or those without access to video technology, frequent personalized telephone calls were employed, although physical performance could not be directly evaluated), and Remote nutritional and psychological interventions (Maintaining nutritional status was prioritized, even over achieving improvements, to prevent deterioration during isolation) (41). Psychological support was similarly offered remotely.

Implementing these solutions during the health crisis required overcoming significant resource limitations, especially regarding personnel and healthcare prioritization. Health systems redirected many professionals to frontline COVID-19 care, reducing human resources available for prehabilitation programs. The drastic reduction in elective surgeries also resulted in fewer interactions with preoperative patients. Consequently, methods had to be more flexible (e.g., telephone follow-up). Adherence to remote programs depended heavily on individual motivation and available support. Without the structure of in-person interactions, some patients showed reduced task compliance (41).

As of now, there is insufficient scientific evidence to definitively recommend a specific anesthetic technique for elderly patients. In our study, locoregional anesthesia techniques were utilized for all participants. The median dose of hyperbaric bupivacaine 0.5% administered was 9 mg +/− 2. In 2019, an average dose of 9 mg +/− 3 of 0.5% hyperbaric bupivacaine was administered, while in 2020, it was 10 mg +/− 1. Notably, peripheral nerve blockade was performed on 76% of patients in 2019 compared to 50% in 2020. Although these differences were not statistically significant, they suggest that during the SARS-CoV-2 pandemic, anesthesiologists administered higher doses of 0.5% intradural hyperbaric bupivacaine and performed fewer peripheral nerve blocks.

The choice of anesthetic technique in elderly patients is crucial due to the physiological changes associated with aging and the presence of comorbidities. Hyperbaric bupivacaine is a common option in regional anesthesia, as it provides effective sensory blockade with less haemodynamic impact than general anesthesia. Various studies, including recent meta-analyses, suggest that regional anesthesia may reduce intraoperative blood loss, shorten surgical duration, and decrease hospital stay compared to general anesthesia. Additionally, it contributes to reduced opioid use and, in some cases, a lower surgical stress response, which is particularly beneficial for patients with cardiovascular and respiratory conditions. On the other hand, while general anesthesia allows for more precise control over the depth and duration of anesthesia, it has been associated with a higher risk of postoperative complications in elderly patients, such as postoperative delirium. However, recent evidence does not show significant differences in 30-day mortality between the two techniques. The choice between regional and general anesthesia should be based on individual factors, such as the patient’s comorbidities and preoperative functional status, as well as the specific contraindications of each technique (42). Thus, there arises a necessity for large-scale randomized controlled clinical trials to provide future guidance on the optimal anesthetic technique for hip surgery in elderly patients. A multidisciplinary treatment and management algorithm are imperative to achieve the best perioperative outcomes.

Given the limitations inherent in this study, it is important to acknowledge that it is observational and retrospective in nature. The susceptibility to biases is high in such studies, primarily influenced by challenges in data collection and the quality of available information. Additionally, the small number of patients included in the study, while ensuring considerable homogeneity through strict inclusion criteria, negatively impacts the statistical power to detect significant differences.

## 5 Conclusion

Regarding the days of hospital admission, it was found that during the year 2020, the length of stay was shorter. Despite the reduction in postoperative hospital surveillance time, no increase in complications was observed. This likely facilitated the patient’s early return to daily life and early functional rehabilitation, while minimizing the likelihood of nosocomial infections minimizing economic costs. A well-structured discharge plan, combined with close follow-up and comprehensive education on post-operative care, can enhance functional outcomes and improve patient and family satisfaction.

In this study, the surgical delay times in both the 2019 and 2020 periods met current recommendations (<48 h). Overall mortality was consistent with previous studies, reaching 22.81%. When separating and studying mortality in the selected groups, no significant differences were found between patients who underwent surgery in both periods.

The anesthetic variables considered in the study showed that survival was better for patients who did not require vasoactive drugs during surgery to maintain blood pressure levels above 90/60 mmHg, compared to those who did need them. It was demonstrated that for each additional point on the Clavien-Dindo scale, the annual mortality rate increased by 2.5 times. Similarly, for each point decrease in hemoglobin levels in the post-surgical blood analysis at 48 h compared to the preoperative level, the mortality rate was 1.6 times higher.

## Data Availability

The data analyzed in this study is subject to the following licenses/restrictions: data were obtained through the review of medical records, ensuring the anonymization of personal information. Requests to access these datasets should be directed to HCU Lozano Blesa.
