# Supplementary material for: Epidemiology and factors associated with the perioperative course of patients undergoing hip fracture during the initial phase of the state of emergency declared in 2020
Source: Front Med (Lausanne). 2025 May 12;12:1473619. doi: 10.3389/fmed.2025.1473619 (PMC12104295; doi:10.3389/fmed.2025.1473619)
Supplement: Supplementary file 1 [file Data_Sheet_1.docx]

# Annex A: Abbreviated Charlson Comorbidity Index (CCI). Interpretation of results.

**Charlson Comorbidity Index (abbreviated)**

Cerebral vascular disease… 1

Diabetes… 1

COPD… 1

Heart failure/ischemic heart disease. 1

Dementia… 1

Peripheral arterial disease… 1

Chronic kidney failure (dialysis)… 2

Cancer… 2

**Annex A:** In general, the absence of comorbidity is indicated by a score of 0-1, low comorbidity by a score of 2 points, and high comorbidity by a score of 3 or more points. Its prognostic utility is similar to that of the original full version.
