# Supplementary material for: Epidemiology and factors associated with the perioperative course of patients undergoing hip fracture during the initial phase of the state of emergency declared in 2020
Source: Front Med (Lausanne). 2025 May 12;12:1473619. doi: 10.3389/fmed.2025.1473619 (PMC12104295; doi:10.3389/fmed.2025.1473619)
Supplement: Supplementary file 2 [file Data_Sheet_2.docx]

# Annex B: ASA Anesthetic Risk Scale

| ASA I | Healthy patient. |
| --- | --- |
| ASA II | Mild systemic disease, without limitations in daily activities (compensated) |
| ASA III | Serious systemic illness that limits your activity, but does not incapacitate your ordinary life (decompensation). |
| ASA IV | Disabling systemic disease that is a constant threat to life. |
| ASA V | Moribund patient, who is not expected to survive more than 24 hours with or without surgical intervention. |
| ASA VI | Brain death. |

**Annex B:** ASA classification is based on the patient's comorbidities. It is important to note that the type of surgery does not influence the score obtained.
