# Supplementary material for: Epidemiology and factors associated with the perioperative course of patients undergoing hip fracture during the initial phase of the state of emergency declared in 2020
Source: Front Med (Lausanne). 2025 May 12;12:1473619. doi: 10.3389/fmed.2025.1473619 (PMC12104295; doi:10.3389/fmed.2025.1473619)
Supplement: Supplementary file 3 [file Data_Sheet_3.docx]

# Annex C: Clavien and Dindo Classification

| Grade I | Any deviation from the normal postoperative period that does not require open or endoscopic reintervention. It is considered to include the use of electrolyte solutions, antiemetics, antipyretics, analgesics and physiotherapies. Includes superficial infection treated in the patient's bed. |
| --- | --- |
| Grade II | Pharmacological treatment different from the previous ones is required. Use of blood transfusions or blood products and parenteral nutrition. |
| Grade III | Requires surgical, endoscopic or radiological reintervention.   1. Without general anesthesia 2. With general anesthesia |
| Grade IV | Complications that threaten the patient's life and require treatment in intermediate or intensive care.   1. Single organic dysfunction. 2. Multiple organ dysfunction. |
| Grade V | Death of the patient. |

**Annex C:** Classification based on the severity of postoperative complications.
